# Lipoteichoic Acid Inhibits *Staphylococcus aureus* Biofilm Formation

**DOI:** 10.3389/fmicb.2018.00327

**Published:** 2018-02-27

**Authors:** Ki Bum Ahn, Jung Eun Baik, Cheol-Heui Yun, Seung Hyun Han

**Affiliations:** ^1^Department of Oral Microbiology and Immunology, DRI, and BK21 Plus Program, School of Dentistry, Seoul National University, Seoul, South Korea; ^2^Research Division for Biotechnology, Korea Atomic Energy Research Institute, Jeongeup, South Korea; ^3^Department of Agricultural Biotechnology and Research Institute of Agriculture and Life Sciences, Seoul National University, Seoul, South Korea

**Keywords:** lipoteichoic acid, *Lactobacillus plantarum*, *Staphylococcus aureus*, biofilm formation, infectious diseases

## Abstract

A biofilm is an aggregate of microorganisms in which cells adhere to biological or non-biological surfaces and is responsible for various infectious diseases. Infections caused by *Staphylococcus aureus*, including pneumonia, endocarditis, and osteomyelitis, are often associated with colonization and biofilm formation. Although lipoteichoic acid (LTA) is involved in biofilm formation, the specific role of LTA is not clearly understood. In this study, we demonstrated that LTA released from *Lactobacillus plantarum* could inhibit *S. aureus* biofilm formation and aggregation without affecting the growth of *S. aureus* in various *in vitro* and *in vivo* models. *L. plantarum* LTA (Lp.LTA) also inhibited biofilm formation of *S. aureus* clinical isolates, including a methicillin-resistant strain. Remarkably, Lp.LTA not only interfered with *S. aureus* biofilm formation, but it also disrupted a pre-formed biofilm. Mechanism studies demonstrated that Lp.LTA inhibited expression of the *ica*-operon, which is responsible for the production of poly-*N*-acetylglucosamine, a key molecule required for *S. aureus* biofilm development. Lp.LTA increased the release of autoinducer-2 from *S. aureus*, which contributed to the inhibition of *S. aureus* biofilm formation. Moreover, Lp.LTA treatment enhanced susceptibility of the biofilm to various antibiotics and to macrophages. Interestingly, Lp.LTA without D-alanine moieties was not able to inhibit biofilm formation by *S. aureus*. In conclusion, the present study suggests that LTA can inhibit *S. aureus* biofilm formation, and therefore could be applied for preventing and/or treating infectious diseases caused by *S. aureus* biofilms.

## Introduction

A biofilm is a community of microorganisms that attaches to biological and non-biological surfaces, including various host tissues (such as lung, intestine, heart valve, and tooth) and indwelling medical devices (such as catheters). Attached microorganisms produce extracellular polymeric substances (EPSs) where they are embedded. Biofilms have been implicated in more than 80% of human microbial infectious diseases, including endocarditis, sinusitis, gingivitis, cystic fibrosis, urinary tract infections, and osteomyelitis (Costerton et al., 1999). Once forming a biofilm, bacteria are 10–1,000 times more resistant to antimicrobial agents than planktonic cells (Mah and O’Toole, 2001) and has the ability to avoid phagocytosis by macrophages and neutrophils (Thurlow et al., 2011; Domenech et al., 2013), leading to recurrent infections or chronic inflammation.

*Staphylococcus aureus* is a Gram-positive pathogen that can cause pneumonia, endocarditis, and septic shock (Lowy, 1998). Approximately 20–30% of healthy humans are permanently colonized with *S. aureus*, while 30% are intermittently colonized (Otto, 2010). Biofilm formation by *S. aureus* increases the emergence of antibiotic-resistant bacteria, such as methicillin-resistant (MRSA) and vancomycin-resistant *S. aureus* (VRSA), which is associated with chronic infection (Davies, 2003). Moreover, multidrug-resistant *S. aureus* has become a public health concern because it leaves only a few treatment options and increases morbidity and mortality. Therefore, effective therapeutic strategies to suppress and prevent the formation of biofilm by *S. aureus* are more than necessary.

Recently, natural anti-biofilm agents have been developed as alternatives to antibiotics to protect against biofilm-related infections. These molecules inhibit biofilm formation without causing the emergence of bacterial resistance and enhance the mode of action for treatment impact of antibiotics. Bacteria-derived amphiphiles have been extensively studied for their anti-biofilm properties. For example, biosurfactants like lipopeptide from *Bacillus subtilis* and glycolipid from *Pseudomonas aeruginosa* inhibit pathogenic bacterial biofilms (Mireles et al., 2001; Rodrigues et al., 2006). In addition, since *Lactobacillus* strains are reported to have beneficial effects without side effects, studies on *Lactobacillus*-derived molecules as anti-biofilm agents have been actively pursued. Culture supernatant from *Lactobacillus acidophilus* inhibits biofilm formation of *S. aureus* (Walencka et al., 2008), and *Lactobacillus*-derived biosurfactants inhibit *Candida albicans* biofilm formation (Ceresa et al., 2015). Thus, bacteria-derived anti-biofilm molecules, especially those from *Lactobacillus*, are considered to be alternative options to overcome increasing antibiotic resistance.

Among the cell wall components of Gram-positive bacteria, lipoteichoic acid (LTA) is a major constituent that has various roles in bacterial division, separation, host recognition, and biofilm formation (Percy and Grundling, 2014; Kang et al., 2016). LTA is an amphiphilic glycolipid linked to a hydrophilic polyphosphate polymer and passes through the cell wall, where it is exposed on the cell surface. So far, five types of LTA (i.e., types I–V) have been identified. Among them, type I LTA has been the most and best characterized that is found in most Gram-positive bacteria including *S. aureus, Streptococcus agalactiae, Streptococcus pyogenes, Bacillus subtilis*, and *Lactobacillus plantarum* (Kang et al., 2016). Type I LTA contains polyglycerol phosphate backbone (Gro-P) linked to glycolipid anchor, dihexosyl-diacylglycerol or dihexosyl-triacylglycerol (Kang et al., 2016). LTA is continuously released during bacterial growth. LTA is recognized by Toll-like receptor 2 (TLR2), leading to the regulation of innate immune responses and further development of adaptive immunity in human and animal (Kang et al., 2016). It has been reported that LTA from *S. aureus* induces inflammatory responses (Kengatharan et al., 1998). In contrast, *L. plantarum* LTA (Lp.LTA) was recently shown to regulate the immune response through inhibiting adhesion of pathogens to host cells, thereby suppressing inflammatory activity (Gao et al., 2016). These conflicting effects of various LTAs are due to their structural differences among different bacterial species (Ryu et al., 2009; Kang et al., 2016).

It has been reported that the LTA plays an important role in colonization of *S. aureus* and *Enterococcus faecalis* (Gross et al., 2001; Fabretti et al., 2006). Moreover, LTA lacking in *S. aureus* alters the hydrophobicity of the bacterial surface resulting in reduction of biofilm formation (Fedtke et al., 2007) and *S. aureus* mutant strain lacking D-alanine, a functional moiety of LTA, reduces the ability to adhere to nasal epithelial cells and its colonization (Weidenmaier et al., 2004). Thus, it is likely that LTA released from bacteria may also regulate biofilm formation. However, the effect of LTA on the biofilm formation of pathogens has rarely been studied. In the present study, we investigated whether Lp.LTA could regulate biofilm formation in *S. aureus*, including the underlying molecular mechanisms, using *in vitro* and *in vivo* biofilm models.

## Materials and Methods

### Bacteria and Reagents

*L. plantarum* KCTC10887BP was obtained from the Korean Collection for Type Culture (Daejeon, Korea). *S. aureus* ATCC 29213, *Streptococcus pneumoniae* ATCC 27336, *E. faecalis* ATCC 29212, and *B. subtilis* ATCC 6633 were obtained from the American Type Culture Collection (Manassas, VA, United States). *Streptococcus gordonii* CH1 was obtained from Professor Paul M. Sullam (University of California at San Francisco). *Vibrio harveyi* BB170 was obtained from Professor Bong-Kyu Choi (Seoul National University, Seoul, Korea). *S. aureus* RN4220 was obtained from Professor Bok Luel Lee (Pusan National University, Pusan, Korea). Clinical isolates of *S. aureus* NCCP14780 and MRSA NCCP14769 were obtained from the National Culture Collection for Pathogens (Osong, Korea). USA300, USA300Δ*luxS*, and USA300Δ*agrA* were obtained from the Nebraska Transposon Mutant Library (Omaha, NE, United States). The Texas Red conjugate of concanavalin A (Texas Red-Con A) and LIVE/DEAD Baclight Bacterial Viability Kit were purchased from Molecular Probes (Eugene, OR, United States). Proteinase K and octyl-sepharose beads were purchased from Sigma–Aldrich Chemical Inc. (St. Louis, MO, United States). DNase I was purchased from Roche Molecular Biochemicals (Laval, QC, Canada).

### Purification of LTA

LTA was prepared from *S. aureus, S. pneumoniae, S. gordonii, E. faecalis, B. subtilis*, and *L. plantarum* as previously described (Ryu et al., 2009). Biologically active molecules such as endotoxins, nucleic acids, and proteins were not detected in the purified LTA preparations (Baik et al., 2008; Ryu et al., 2009; Kim et al., 2017). Structural intactness of LTA was confirmed with high-field nuclear magnetic resonance and matrix-assisted laser desorption ionization-time of flight mass spectrometry, as previously described (Jang et al., 2011). In some experiments, LTA was further treated with proteinase K (50 μg/ml) or DNase I (50 μg/ml) at 37°C for 1 h, or heat at 100°C for 10 min. D-Alanine moieties-removed Lp.LTA was prepared by incubating intact Lp.LTA with 0.1 M Tris-HCl at pH 8.5 for 24 h, as previously described (Baik et al., 2011). Lp.LTA lacking both D-alanine and acyl moieties was prepared by incubating intact Lp.LTA in 0.5 N NaOH for 2 h, as previously described (Baik et al., 2011).

### Preparation of Culture Supernatant from *L. plantarum*

*L. plantarum* was grown in de Man, Rogosa and Sharpe (MRS) broth at 37°C for 24 h. The culture was centrifuged at 10,410 × *g* for 10 min at 4°C. The culture supernatants were filtered through a 0.2 μm membrane filter to remove the remaining bacteria and debris, and then stored at -20°C. In some experiments, *L. plantarum* culture supernatants (Lp.sup) were further treated with proteinase K (50 μg/ml) or DNase I (50 μg/ml) at 37°C for 1 h, or heat at 100°C for 10 min.

### Isolation of Lipoproteins

Lipoproteins from *L. plantarum* were isolated as described previously (Li et al., 2008). *L. plantarum* was pelleted and resuspended in Tris-buffered saline (TBS) containing protease inhibitors. The bacterial lysate was incubated with 2% Triton X-114 at 4°C for 2 h. After centrifugation at 10,410 × *g* for 10 min, the supernatants were collected and further incubated at 37°C for 15 min. After centrifugation at 10,410 × *g* for 10 min, the aqueous phase was discarded, and an equal volume of TBS was added to the Triton X-114 phase. After incubating at 37°C for 15 min, the Triton X-114 phase was collected by centrifugation at 10,410 × *g* for 10 min and mixed with methanol at -20°C overnight. The precipitated lipoproteins were dissolved in 10 mM octyl β-D-glucopyranoside.

### Crystal Violet Staining

*S. aureus* (5 × 10^7^ CFU/ml) was grown in Luria-Bertani (LB) broth on 96-well plates at 37°C for 24 h in the presence or absence of LTA. Planktonic bacteria were removed by gentle washing twice with phosphate-buffered saline (PBS). Biofilms were stained with 0.1% crystal violet solution for 30 min at room temperature, followed by washes with PBS to remove non-specific stain. The adhering dye was dissolved in solution (95% ethanol and 0.1% acetic acid), and absorbance was measured at 600 nm in a microplate reader (Molecular Devices, Sunnyvale, CA, United States).

### Confocal Laser Scanning Microscopy

*S. aureus* (5 × 10^7^ CFU/ml) was grown in LB broth on glass-bottom dishes at 37°C for 24 h in the presence of Lp.LTA (10, 30, or 50 μg/ml). Planktonic bacteria were removed by gentle washing with PBS. The biofilms were stained with SYTO9 and propidium iodide using the LIVE/DEAD BacLight Bacterial Viability Kit to distinguish live and dead bacteria, respectively. After washing with PBS, the biofilms were visualized by LSM700 confocal laser scanning microscopy (Zeiss, Jena, Germany).

### Scanning Electron Microscope (SEM)

*S. aureus* (5 × 10^7^ CFU/ml) was grown in LB broth on 24-well plates at 37°C for 24 h in the presence of Lp.LTA (10 or 30 μg/ml) and then gently washed with PBS to remove planktonic bacteria. The biofilms were prefixed with PBS containing 2.5% glutaraldehyde and 2% paraformaldehyde at 4°C overnight, and then washed with PBS. The biofilms were subsequently fixed with 1% osmium tetroxide for 1.5 h, then washed three times with distilled water and dehydrated in serially graded ethanol solutions (70, 80, 90, 95, and 100% each for 15 min). After drying with a critical point dryer (HCP-2; Hitachi, Tokyo, Japan) and coating with an ion sputter (Quorum Q150 T S; Quorum Technologies Ltd., East Grinstead, United Kingdom), the samples were observed via SEM (S-4700; Hitachi, Tokyo, Japan).

### Reverse Transcription-Polymerase Chain Reaction (RT-PCR)

RT-PCR was performed as previously described (Atshan et al., 2012; Lee et al., 2015). *S. aureus* (1 × 10^8^ CFU/ml) was stimulated with 10, 30, or 50 μg/ml of Lp.LTA for 3 h. Total RNA was prepared using the easy-RED^TM^ BYF Total RNA Extraction Kit (iNtRON, Sungnam, Korea) and complementary DNA (cDNA) was synthesized from 5 μg of total RNA. Amplification of cDNA by PCR was performed in 20 μl reaction containing 0.5 units of rTaq DNA polymerase and 10 pmol of primers specific to *icaA, icaB, icaC, icaD, clfA, clfB, cna, eno*, and *gyrB* (**Table 1**) for 35 cycles. Amplified PCR products were separated on 1.5% agarose gels and visualized by staining with ethidium bromide.

**Table 1:** Primer pairs for amplification of PCR target genes.

Gene	Primer sequence
*icaA* (intercellular adhesion gene A)	Forward: 5′-ACACTTGCTGGCGCAGTCAA-3′
	Reverse: 5′-TCTGGAACCAACATCCAACA-3′
*icaB* (intercellular adhesion gene B)	Forward: 5′-AGAATCGTGAAGTATAGAAAATT-3′
	Reverse: 5′-TCTAATCTTTTTCATGGAATCCGT-3′
*icaC* (intercellular adhesion gene C)	Forward:5′ATGGGACGGATTCCATGAAAAAGA-3′
	Reverse: 5′-TAATAAGCATTAATGTTCAATT-3′
*icaD* (intercellular adhesion gene D)	Forward: 5′-ATGGTCAAGCCCAGACAGAG-3′
	Reverse: 5′-AGTATTTTCAATGTTTAAAGCAA-3′
*clfA* (clumping factor A)	Forward: 5′-ATTGGCGTGGCTTCAGTGCT-3′
	Reverse: 5′-CGTTTCTTCCGTAGTTGCATTTG-3′
*clfB* (clumping factor B)	Forward: 5′-ACATCAGTAATAGTAGGGGCAAC-3′
	Reverse: 5′-TTCGCACTGTTTGTGTTTGCAC-3′
*cna* (collagen binding protein)	Forward: 5′-AAAGCGTTGCCTAGTGGAGA-3′
	Reverse: 5′-AGTGCCTTCCCAAACCTTTT-3′
*eno* (laminin binding protein)	Forward: 5′-ACGTGCAGCAGCTGACT-3′
	Reverse: 5′-CAACAGCATCTTCAGTACCTTC-3′
*gyrB* (gyrase B)	Forward: 5′-TTATGGTGC TGGACAGATACA-3′
	Reverse: 5′-CACCGTGAAGACCGCCAGATA-3′

### Autoinducer-2 (AI-2) Measurement

The AI-2 reporter assay was performed as previously described (Bassler et al., 1993; Schauder et al., 2001). Briefly, *S. aureus* (1 × 10^7^ CFU/ml) was grown in the absence or presence of Lp.LTA (10, 30, or 50 μg/ml) at 37°C for 24 h and 20 μl of *S. aureus* culture supernatants were mixed with 180 μl of the AI-2 reporter strain, *V. harveyi* BB170. Luminescence was measured using a GloMax microplate luminometer (Promega, WI, United States).

### Macrophage-Biofilm Interaction Assay

The experiment was done as previously described, with slight modifications (Chua et al., 2014). Briefly, *S. aureus* (5 × 10^7^ CFU/ml) was grown in LB broth on sterile glass coverslips at 37°C for 24 h in the presence or absence of Lp.LTA (30 μg/ml). The biofilm was washed with PBS to remove planktonic bacteria. A murine macrophage cell line, RAW 264.7, was added at 1 × 10^6^ cells/ml to the *S. aureus* biofilm and incubated at 37°C for 2 h in a humidified incubator with 5% CO_2_. The biofilm was washed with PBS to remove RAW 264.7 cells and dispersed bacteria. Then, the remaining biofilm was harvested and suspended in PBS. The samples were diluted and plated on LB agar plates, and the plates were incubated at 37°C for 24 h. After incubation, the number of bacterial colonies was counted.

### *In Vivo* Biofilm Model

Experiments using a mouse model in the study were approved by the Institutional Animal Care and Use Committee of Seoul National University (SNU-170518-5). A catheter lumen was inoculated with *S. aureus* (5 × 10^7^ CFU/ml) in the presence or absence of Lp.LTA (100 μg/ml). Then, the catheters were implanted subcutaneously into the dorsal areas of 7-week-old female C57BL/6 mice after anesthesia with 125 mg/kg of tribromoethanol (Sigma–Aldrich Chemical Inc.). The mice were sacrificed at day 3 post-infection to harvest the catheters. The catheters were sonicated in PBS for 10 min to obtain the biofilm bacteria from the catheters. Each sample was diluted and plated on LB agar plates, and the plates were incubated at 37°C for 24 h. After incubation, the number of bacterial colonies was counted and compared for each sample.

### Statistical Analysis

All experiments were performed at least three times. The mean value ± standard deviation (SD) was obtained from triplicate samples for each treatment group. Statistical significance was examined with a *t*-test. Asterisks indicate significant induction compared with the non-treatment group (^∗^*P* < 0.05, ^∗∗^*P* < 0.01 and ^∗∗∗^*P* < 0.001).

## Results

### *L. plantarum* LTA Has Inhibitory Effects on *S. aureus* Biofilm Formation

**FIGURE 1:**
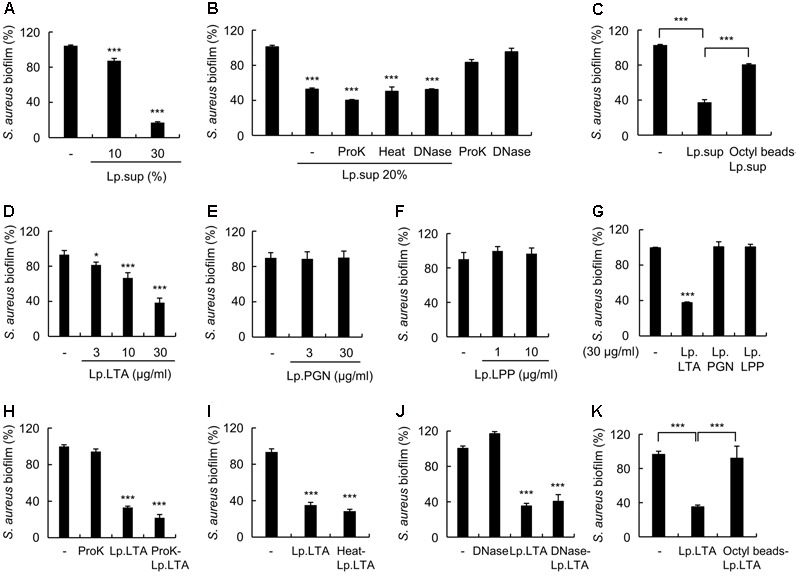
*L. plantarum* LTA has inhibitory effects on *S. aureus* biofilm formation. *S. aureus* (5 × 10^7^ CFU/ml) was grown on polystyrene plates at 37°C for 24 h in the presence of **(A)** *L. plantarum* culture supernatants (Lp.sup) (10 or 30%), **(B)** 50 μg/ml of proteinase K (ProK)-treated Lp.sup (20%), heat-treated Lp.sup (20%) for 10 min at 100°C, or 50 μg/ml of DNase I (DNase)-treated Lp.sup (20%), **(C)** octyl-sepharose bead-treated Lp.sup (20%), **(D)** *L. plantarum* LTA (Lp.LTA) (3, 10, or 30 μg/ml), **(E)** *L. plantarum* peptidoglycan (Lp.PGN) (3 or 30 μg/ml), **(F)** *L. plantarum* lipoprotein (Lp.LPP) (1 or 10 μg/ml), **(G)** *S. aureus* (5 × 10^7^ CFU/ml) was grown on sterile glass coverslips at 37°C for 24 h in the presence of Lp.LTA (30 μg/ml), Lp.PGN (30 μg/ml), or Lp.LPP (30 μg/ml). *S. aureus* (5 × 10^7^ CFU/ml) was grown on polystyrene plates at 37°C for 24 h in the presence of 30 μg/ml of Lp.LTA or **(H)** 50 μg/ml of proteinase K-treated Lp.LTA (30 μg/ml), **(I)** heat-treated Lp.LTA (30 μg/ml), **(J)** 50 μg/ml of DNase I-treated Lp.LTA (30 μg/ml), or **(K)** octyl-sepharose bead-treated Lp.LTA (30 μg/ml). The extent of biofilm formation was determined via crystal violet assay. Data are the mean values ± SD of triplicate samples. Asterisks indicate significant induction compared with the non-treatment group (^∗^*P* < 0.05 and ^∗∗∗^*P* < 0.001).

It has been reported that culture supernatants from *Lactobacillus* strains inhibit the biofilm formation of various pathogenic bacteria (Soderling et al., 2011; Melo et al., 2016). We first examined whether Lp.sup could inhibit *S. aureus* biofilm formation. As shown in **Figure 1A**, Lp.sup inhibited the biofilm formation of *S. aureus* in a dose-dependent manner. To characterize the molecules responsible for Lp.sup-mediated inhibition of biofilm formation, *S. aureus* was treated with Lp.sup, proteinase K (ProK)-treated Lp.sup, DNase I (DNase)-treated Lp.sup, heat-treated Lp.sup, or octyl-sepharose bead-treated Lp.sup. As shown in **Figure 1B**, proteinase K, DNase I, and heat treatment did not significantly alter the inhibitory effects of Lp.sup, whereas octyl-sepharose bead-treated Lp.sup led to significant recovery of the inhibitory activity of the Lp.sup on biofilm formation of *S. aureus* (**Figure 1C**). These results suggest that hydrophobic molecules are an essential component in the Lp.sup that inhibits biofilm formation by *S. aureus*. To identify the molecule in Lp.sup that inhibited *S. aureus* biofilm formation, we examined biofilm formation of *S. aureus* after treatment with representative microbe-associated molecular patterns, including Lp.LTA, *L. plantarum* PGN (Lp.PGN), and *L. plantarum* lipoproteins (Lp.LPP). As shown in **Figures 1D–G**, Lp.LTA inhibited *S. aureus* biofilm formation in a dose-dependent manner on either polystyrene or glass surface, whereas Lp.PGN and Lp.LPP did not inhibit biofilm formation. To confirm that Lp.LTA is a major component of Lp.sup inhibiting biofilm formation, *S. aureus* biofilm formation was examined in the presence of proteinase K-treated Lp.LTA, heat-treated Lp.LTA, DNase I-treated Lp.LTA, or octyl-sepharose bead-treated Lp.LTA. Concordant with **Figures 1B,C**, proteinase K, heat, and DNase I treatment did not significantly alter the inhibitory effects of Lp.LTA on biofilm formation of *S. aureus*, whereas octyl-sepharose bead-treated Lp.LTA significantly recovered the inhibition by Lp.LTA (**Figures 1H–K**). These results suggest that Lp.LTA is the likely candidate in Lp.sup responsible for the inhibition of *S. aureus* biofilm formation.

### *L. plantarum* LTA Inhibits Biofilm Formation and Aggregation of *S. aureus in Vitro* and *in Vivo*

**FIGURE 2:**
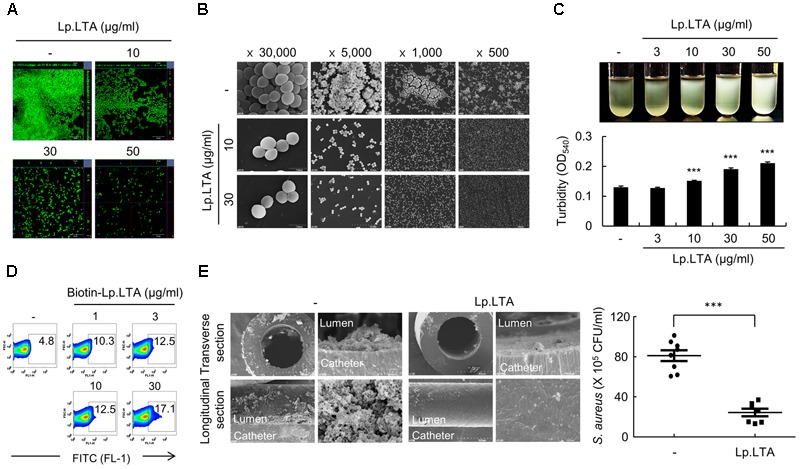
*L. plantarum* LTA inhibits biofilm formation and aggregation of *S. aureus in vitro* and *in vivo*. **(A,B)** *S. aureus* (5 × 10^7^ CFU/ml) was grown on polystyrene plates at 37°C for 24 h in the absence or presence of Lp.LTA (10, 30, or 50 μg/ml). Lp.LTA-treated *S. aureus* biofilms were visualized **(A)** by confocal laser scanning microscopy (green from SYTO9 and red from propidium iodide) or **(B)** by scanning electron microscopy (Magnification: ×500, ×1,000, ×5,000, or ×30,000). **(C)** *S. aureus* (2 × 10^8^ CFU/ml) was grown for 24 h in the absence or presence of 3, 10, 30, or 50 μg/ml of Lp.LTA. Aggregation of *S. aureus* was determined by optical density (O.D.) at 540 nm. Data are the mean values ± SD of triplicate samples. **(D)** *S. aureus* (5 × 10^7^ CFU/ml) was treated with 1, 3, 10, or 30 μg/ml of biotinylated Lp.LTA (biotin-Lp.LTA) for 1 h. Binding of biotin-Lp.LTA with *S. aureus* was detected by streptavidin-FITC and analyzed via flow cytometry. The percentage of biotin-Lp.LTA-positive *S. aureus* is shown in each histogram. One of three similar results is shown. **(E)** C57BL/6 mice were implanted with *S. aureus*-loaded catheter in the presence or absence of Lp.LTA. After 3 days, the mice were sacrificed to harvest the catheters. The catheters were sectioned to obtain transverse rings and longitudinal strips, which were subject to SEM analysis (Magnification: ×100, ×2,000, ×5,000, and ×100, clockwise in each treatment group), and the remaining biofilms on the catheters were measured by counting colony forming units. Eight and seven mice were used in the non-treatment and LpLTA-treatment groups, respectively. Asterisks indicate significant induction compared with the non-treatment group (^∗∗∗^*P* < 0.001).

Next, the *S. aureus* biofilm formed in the presence of Lp.LTA was evaluated visually and quantitatively using various experimental models. Confocal microscopy indicated that *S. aureus* biofilm formation was inhibited by Lp.LTA, whereas dead cells (i.e., PI-stained) were hardly made by Lp.LTA (**Figure 2A**). SEM analysis indicated that Lp.LTA inhibited the attachment and aggregation of *S. aureus* on the culture plate but did not induce any morphological changes (**Figure 2B**). In addition, Lp.LTA dose-dependently inhibited the aggregation of *S. aureus* in liquid culture media (**Figure 2C**). Next, the ability of Lp.LTA to bind *S. aureus* was examined. *S. aureus* was treated with biotinylated-LTA, and its binding to *S. aureus* was measured by flow cytometry using streptavidin-FITC. **Figure 2D** shows that the binding of Lp.LTA to *S. aureus* increased in a dose-dependent manner, implying a direct binding of Lp.LTA to *S. aureus*. To examine whether Lp.LTA inhibits biofilm formation of *S. aureus in vivo*, we used a mouse model where a catheter loaded with *S. aureus* in the presence or absence of Lp.LTA was implanted subcutaneously into the mouse dorsal area. After 3 days, catheters were taken from the mice to enumerate the biofilm bacteria. As shown in **Figure 2E**, Lp.LTA treatment substantially reduced the biofilm bacterial load on the catheter compared with the non-treatment control group. These results suggest that Lp.LTA could be used clinically to treat *S. aureus* biofilm-associated infectious diseases.

### *L. plantarum* LTA Inhibits Biofilm Development at Both Early and Late Stages, and Can Disrupt Pre-formed Biofilm

**FIGURE 3:**
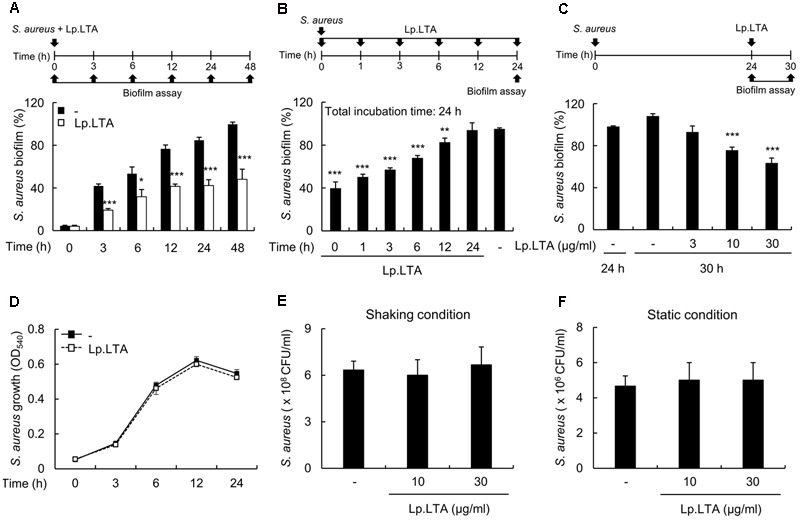
*L. plantarum* LTA inhibits biofilm development at early and late stages, and even disrupts the pre-formed biofilm. **(A)** *S. aureus* (1 × 10^7^ CFU/ml) was grown on polystyrene plates at 37°C for 3, 6, 12, 24, or 48 h in the presence or absence of Lp.LTA (30 μg/ml). **(B)** *S. aureus* (5 × 10^7^ CFU/ml) was grown on polystyrene plates at 37°C for 1, 3, 6, or 12 h, followed by treatment with Lp.LTA (30 μg/ml) at 37°C for up to 24 h. **(C)** *S. aureus* (5 × 10^7^ CFU/ml) was grown on polystyrene plates at 37°C for 24 h, and then supernatant containing planktonic bacteria was removed. Pre-formed biofilm was treated with Lp.LTA (3, 10, or 30 μg/ml) and further incubated at 37°C for 6 h. Biofilm formation was determined by a crystal violet assay. **(D)** *S. aureus* (1 × 10^7^ CFU/ml) was grown under shaking conditions for 3, 6, 12, or 24 h in the presence or absence of Lp.LTA (30 μg/ml). The growth of *S. aureus* was determined by O.D. at 540 nm. **(E,F)** *S. aureus* (5 × 10^7^ CFU/ml) was grown in the presence or absence of Lp.LTA (10 or 30 μg/ml) for 24 h under **(E)** shaking or **(F)** static culture condition. Bacterial viability was measured by counting colony forming units. Data are the mean values ± SD of triplicate samples. Asterisks indicate significant differences compared with the non-treatment group (^∗^*P* < 0.05, ^∗∗^*P* < 0.01, and ^∗∗∗^*P* < 0.001).

To examine the phase of biofilm development at which the inhibitory activity of Lp.LTA occurs, *S. aureus* biofilm formation at 0, 3, 6, 12, 24, and 48 h was measured after treatment with Lp.LTA. Lp.LTA inhibited biofilm formation at all time points examined (**Figure 3A**), without alteration of bacterial growth and viability (**Figures 3D–F**). In order to determine the time point at which the inhibitory effect of LTA occurs during biofilm development, *S. aureus* pre-incubated for 0, 1, 3, 6, 12, or 24 h was treated with Lp.LTA, and biofilm formation was measured at 24 h. **Figure 3B** shows that Lp.LTA inhibited biofilm formation during the treatment period until 12 h. Next, we examined whether Lp.LTA could destroy a pre-formed biofilm of *S. aureus*. For biofilm formation, *S. aureus* was incubated for 24 h, and then the supernatant containing planktonic bacteria was removed. The pre-formed biofilm was treated with Lp.LTA for 6 h, and the degree of destruction on the pre-formed biofilm was examined. As shown in **Figure 3C**, Lp.LTA destroyed the pre-formed biofilm of *S. aureus* in a dose-dependent manner. These results suggest that Lp.LTA inhibits *S. aureus* biofilm formation at early and late stages without affecting bacterial growth, and even disrupts pre-existing biofilms.

### *L. plantarum* LTA Inhibits the Biofilm Formation of Various Strains of *S. aureus*, and Purified LTAs from Various Gram-Positive Bacteria Also Have an Inhibitory Effect

**FIGURE 4:**
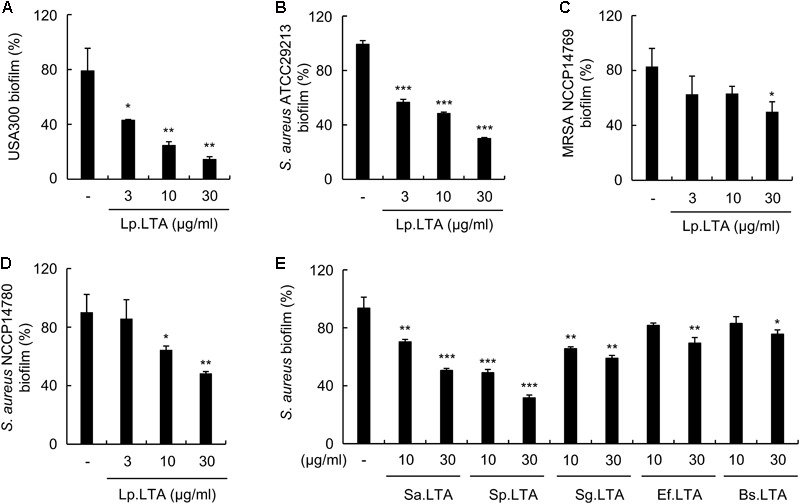
*L. plantarum* LTA inhibits biofilm formation in various strains of *S. aureus*, and purified LTAs from various Gram-positive bacteria also have an inhibitory effect. **(A)** *S. aureus* USA300, **(B)** ATCC 29213, **(C)** NCCP14769, or **(D)** NCCP14780 were treated with 3, 10, or 30 μg/ml of Lp.LTA for 24 h. **(E)** *S. aureus* (5 × 10^7^ CFU/ml) was grown on polystyrene plates at 37°C for 24 h in the presence of purified LTA from *S. aureus, S. pneumoniae, S. gordonii, E. faecalis*, or *B. subtilis.* Biofilm formation was determined via crystal violet assay. Data are the mean values ± SD of triplicate samples. Asterisks indicate significant inhibition compared with the non-treatment group (^∗^*P* < 0.05, ^∗∗^*P* < 0.01, and ^∗∗∗^*P* < 0.001).

In order to determine whether Lp.LTA inhibits both laboratory and clinical strains, the biofilm formation of USA300, ATCC 22913, and clinical isolates such as NCCP14780 and NCCP14769 was measured after treatment with Lp.LTA. As shown in **Figure 4**, Lp.LTA inhibited the biofilm formation of all *S. aureus* strains tested regardless of laboratory and clinical strains. These results indicate that the inhibitory effect of Lp.LTA is not limited to a particular *S. aureus* strain. LTA is present in almost all Gram-positive bacterial cell wall, though the LTAs of various Gram-positive bacteria are reported to differ in their structure and function (Kang et al., 2016). Thus, to investigate whether an inhibitory effect on the biofilm formation of *S. aureus* is a shared characteristic of all LTAs, LTA was purified from various bacterial species including *S. aureus, S. pneumoniae, S. gordonii, E. faecalis*, and *B. subtilis*, and their effects on *S. aureus* biofilm formation was examined. As shown in **Figure 4E**, all LTAs tested in this experiment inhibited *S. aureus* biofilm formation, despite differences in the inhibition potency. These results suggest that inhibition of *S. aureus* biofilm formation is a common characteristic of Gram-positive bacterial LTAs.

### *L. plantarum* LTA Induces AI-2 Release, Leading to Down-Regulation of *ica* Gene Expression and Exopolysaccharide Production to Contribute to the Inhibition of *S. aureus* Biofilm Formation

**FIGURE 5:**
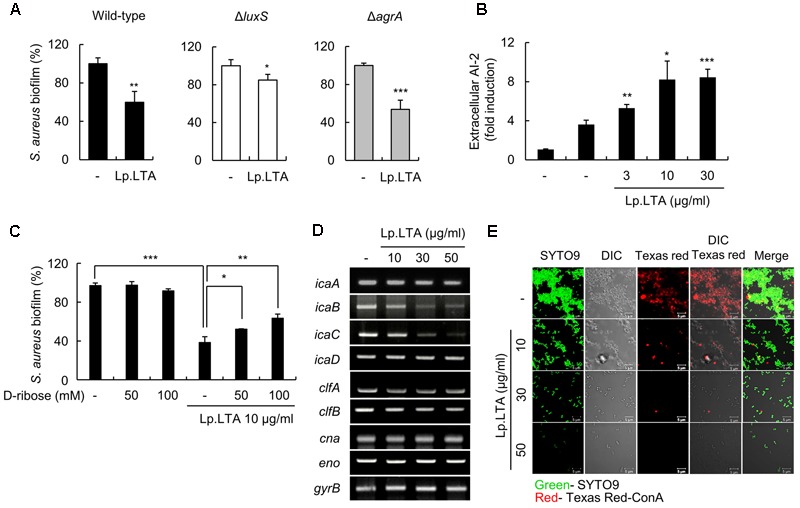
*L. plantarum* LTA induces AI-2 release and down-regulates *ica* gene expression and exopolysaccharide production in *S. aureus*. **(A)** *S. aureus* wild-type (1 × 10^7^ CFU/ml), an AI-2-deficient strain (Δ*luxS*) (1 × 10^7^ CFU/ml), and an AIP-deficient strain (Δ*agrA*) (1 × 10^7^ CFU/ml) were grown in the absence or presence of Lp.LTA (10 μg/ml) at 37°C for 24 h. **(B)** *S. aureus* (1 × 10^7^ CFU/ml) was grown in the absence or presence of Lp.LTA (3, 10, or 30 μg/ml) on polystyrene plates at 37°C for 24 h. The culture supernatants were collected to determine AI-2 release using a bioluminescent bacterial reporter strain, *Vibrio harveyi* BB170. **(C)** *S. aureus* (5 × 10^7^ CFU/ml) was grown on polystyrene plates at 37°C for 24 h in the absence or presence of Lp.LTA (10 μg/ml) with 50 or 100 mM of D-ribose. Biofilm formation was determined via crystal violet assay. Data are the mean values ± SD of triplicate samples. **(D)** *S. aureus* (1 × 10^8^ CFU/ml) was treated with 0, 10, 30, or 50 μg/ml of Lp.LTA for 3 h. Total RNA was isolated, and the mRNA expression of *icaA, icaB, icaC, icaD, clfA, clfB, cna, eno*, and *gyrB* was examined by RT-PCR. **(E)** *S. aureus* (5 × 10^7^ CFU/ml) was grown at 37°C for 24 h in the presence of Lp.LTA (0, 10, 30, or 50 μg/ml). Then, *S. aureus* biofilm formation and exopolysaccharide production were examined with SYTO9 and Texas Red-ConA staining, respectively, followed by analysis using confocal microscopy (green from SYTO9 and red from Texas Red-ConA). Asterisks indicate significant induction compared with the non-treatment group (^∗^*P* < 0.05, ^∗∗^*P* < 0.01, and ^∗∗∗^*P* < 0.001).

Quorum-sensing molecules are critically involved in bacterial biofilm formation. In case of *S. aureus*, AI-2 and AIP are known to inhibit biofilm formation through autocrine pathways (Boles and Horswill, 2008; Yu et al., 2012). To determine whether AI-2 and AIP are involved in the inhibitory effect of Lp.LTA against *S. aureus* biofilm formation, wild-type cells, a Δ*luxS* mutant (an AI-2-deficient strain), and a Δ*agrA* mutant (an AIP-deficient strain) were treated with Lp.LTA, and biofilm formation was measured via crystal violet assay. As shown in **Figure 5A**, the biofilm formation of the wild-type and Δ*agrA* strains was effectively inhibited by Lp.LTA (40.2 and 46.3%, respectively), whereas Δ*luxS* biofilm formation was inhibited by Lp.LTA less dramatically (15.1%). Thus, Lp.LTA inhibition of *S. aureus* biofilm formation is likely to be mediated by AI-2 rather than AIP. Concordantly, Lp.LTA enhanced extracellular AI-2 production in *S. aureus* culture supernatants, as determined by a bioluminescence assay using the *V. harveyi* BB170 bioluminescent reporter strain (**Figure 5B**). Moreover, when *S. aureus* was pretreated with an AI-2 antagonist, D-ribose, the inhibitory effect of Lp.LTA on *S. aureus* biofilm formation was attenuated (**Figure 5C**). These results suggest that AI-2 might play an important role in the inhibition by Lp.LTA of *S. aureus* biofilm formation. Previous reports suggest that extracellular AI-2 inhibits the expression of the intracellular adhesion (*ica*) locus that is essential for the synthesis of the exopolysaccharide poly-*N*-acetylglucosamine (PNAG), a major component of staphylococcal biofilm (O’Gara, 2007; Otto, 2009). Therefore, we examined the effect of Lp.LTA on expression of *ica* locus in *S. aureus*. In addition, the expression of staphylococcal microbial surface components recognizing adhesive matrix molecules (MSCRAMMs) genes, such as *clfA* (encoding clumping factor A), *clfB* (encoding clumping factor B), *cna* (encoding collagen binding protein), and *eno* (encoding laminin binding protein), which mediate initial attachment of staphylococci (Otto, 2008), were examined in the presence of Lp.LTA. As shown **Figure 5D**, Lp.LTA significantly inhibited the mRNA expression of *icaA, icaB*, and *icaC* in *S. aureus*. The mRNA expression of *clfA* and *clfB* was slightly reduced by Lp.LTA, but that of *cna* and *eno* was not reduced. Moreover, Lp.LTA also inhibited PNAG production by *S. aureus* (**Figure 5E**). These observations suggest that Lp.LTA inhibits *ica* gene expression and PNAG production, leading to the inhibition of *S. aureus* biofilm formation.

### D-Alanine Moieties of *L. plantarum* LTA Are Critical for the Inhibitory Effect on *S. aureus* Biofilm Formation

**FIGURE 6:**
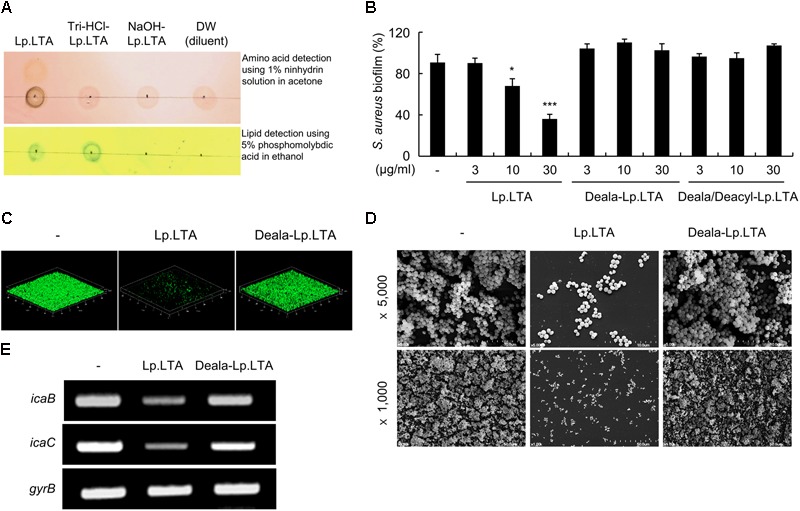
D-Alanine moieties of Lp.LTA are critical for the inhibitory effect on *S. aureus* biofilm formation. **(A)** Dealanylated (Deala)-Lp.LTA and dealanylated/deacylated (Deala/Deacyl)-Lp.LTA were prepared by treatment of Lp.LTA with Tris-HCl (0.1 M, pH 8.5) or NaOH (0.5 N, pH 12), respectively. Then, D-alanine and acyl chain moieties of Lp.LTA were detected by 1% ninhydrin solution and 5% phosphomolybdic acid, respectively. **(B)** *S. aureus* (5 × 10^7^ CFU/ml) was grown on polystyrene plates at 37°C for 24 h in the presence of 0, 3, 10, or 30 μg/ml of Lp.LTA, Deala-Lp.LTA, or Deala/Deacyl-Lp.LTA. Biofilm formation was determined by a crystal violet assay. Data are the mean values ± SD of triplicate samples. **(C)** *S. aureus* (5 × 10^7^ CFU/ml) was grown on glass-bottom dishes at 37°C for 24 h in the absence or presence of 30 μg/ml of Lp.LTA or Deala-Lp.LTA. *S. aureus* biofilms were visualized by confocal laser scanning microscopy (green from SYTO9 and red from propidium iodide). **(D)** *S. aureus* (5 × 10^7^ CFU/ml) was grown at 37°C for 24 h in the absence or presence of 30 μg/ml of Lp.LTA or Deala-Lp.LTA. *S. aureus* biofilms were analyzed by scanning electron microscopy (magnification: ×1,000 or ×5,000). **(E)** *S. aureus* (1 × 10^8^ CFU/ml) was treated with 30 μg/ml of Lp.LTA or Deala-Lp.LTA for 3 h. Total RNA was isolated, and the expression of *icaB, icaC*, and *gyrB* mRNA was examined by RT-PCR. Asterisks indicate significant induction compared with the non-treatment group (^∗^*P* < 0.05 and ^∗∗∗^*P* < 0.001).

D-Alanine moieties-removed Lp.LTA (Deala-Lp.LTA) and Lp.LTA deficient in both D-alanine and acyl moieties (Deala/Deacyl-Lp.LTA) were prepared by treatment with HCl (pH 8.5) and NaOH (pH 12), respectively, as previously described (Baik et al., 2011). Dealanylation and deacylation of Lp.LTA were confirmed by 1% ninhydrin solution and 5% phosphomolybdic acid on silica plates, respectively (**Figure 6A**). To identify the functional moieties responsible for the inhibitory effects of Lp.LTA on biofilm formation, biofilm formation was examined in *S. aureus* after treatment with Lp.LTA, Deala-Lp.LTA, and Deala/Deacyl-Lp.LTA. As shown in **Figure 6B**, Lp.LTA, but not Deala-Lp.LTA or Deala/Deacyl-Lp.LTA, inhibited biofilm formation by *S. aureus* in a dose-dependent manner. Confocal and electron microscopy analyses also indicated that *S. aureus* biofilm formation was inhibited by Lp.LTA, but not by Deala-Lp.LTA (**Figures 6C,D**). Furthermore, the inhibitory effects of Lp.LTA on *ica* mRNA expression were examined in *S. aureus* treated with Lp.LTA or Deala-Lp.LTA. As shown in **Figure 6E**, Lp.LTA inhibited *icaB* and *icaC* mRNA expression, whereas Deala-Lp.LTA failed to do so, indicating that D-alanine is a critical component of Lp.LTA that exerts inhibitory effects on biofilm formation by *S. aureus*.

### *L. plantarum* LTA Increases the Susceptibility of *S. aureus* Biofilms to Antibiotics and Macrophages

**FIGURE 7:**
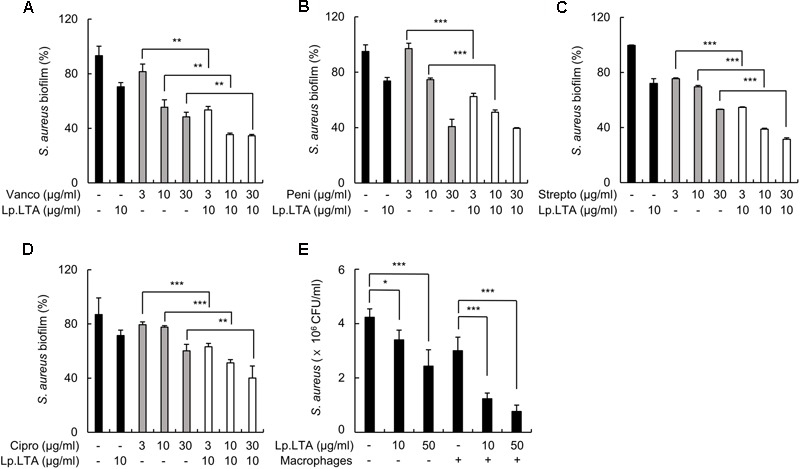
*L. plantarum* LTA increases the susceptibility of *S. aureus* biofilms to antibiotics and macrophages. *S. aureus* (5 × 10^7^ CFU/ml) was grown on polystyrene plates with 10 μg/ml of Lp.LTA at 37°C for 24 h, and further incubated for 6 h in the absence or presence of 3, 10, or 30 μg/ml of antibiotics that target **(A,B)** cell wall synthesis (vancomycin and penicillin), **(C)** protein synthesis (streptomycin), or **(D)** nucleic acid synthesis (ciprofloxacin). The extent of biofilm formation was determined by a crystal violet assay. Data are the mean values ± SD of triplicate samples. **(E)** *S. aureus* (5 × 10^7^ CFU/ml) was grown on sterile glass coverslips at 37°C for 24 h in the presence or absence of Lp.LTA (10 or 50 μg/ml) and treated with RAW 264.7 cells (1 × 10^6^ cells/ml) at 37°C for 2 h. Remaining biofilms were measured by counting colony forming units. Data are the mean values ± SD of triplicate samples. Asterisks indicate significant induction compared with the non-treatment group or the macrophages alone-treatment group (^∗^*P* < 0.05, ^∗∗^*P* < 0.01, and ^∗∗∗^*P* < 0.001).

To examine whether Lp.LTA could enhance the inhibitory effects of antibiotics on *S. aureus* biofilm formation, *S. aureus* was treated with various antibiotics that target cell wall synthesis (vancomycin and penicillin), protein synthesis (streptomycin), or nucleic acid synthesis (ciprofloxacin) in the presence of Lp.LTA. Biofilm formation was then examined. As shown in **Figures 7A–D**, Lp.LTA enhanced the ability of all classes of antibiotics to inhibit biofilm formation by *S. aureus*. In addition, the effect of Lp.LTA on the susceptibility of *S. aureus* biofilms to macrophages was investigated. As shown in **Figure 7E**, Lp.LTA treatment improved the removal of *S. aureus* biofilms by macrophages. Collectively, these results suggest that Lp.LTA potentiates the antimicrobial functions of antibiotics and macrophages.

## Discussion

*S. aureus* is a notorious Gram-positive pathogen that causes various inflammatory diseases, and its biofilm formation is closely associated with chronic infection and antibiotic resistance. In this study, we demonstrated that Lp.LTA efficiently inhibited *S. aureus* biofilm formation in various *in vitro* and *in vivo* experimental models. Mechanistic studies further showed that Lp.LTA abrogated the production of an exopolysaccharide (PNAG, an important component of staphylococcal biofilms) by increasing AI-2 production and down-regulating the expression of *ica* genes. Our results provide the proof-of-concept that Lp.LTA is a potential standalone antimicrobial agent, or a potential additive to antibiotics to potentiate their antimicrobial activities.

The inhibitory effect on *S. aureus* biofilm formation seems not to be unique to Lp.LTA, but is more likely a common feature of the LTAs of most Gram-positive bacteria. Indeed, we also observed such inhibitory effects by the LTAs purified from other Gram-positive bacteria including *S. pneumoniae, S. gordonii, E. faecalis*, and *B. subtilis*. This result is coincident with previous reports, such as the inhibition of *Streptococcus mutans* colonization by *S. mutans* LTA (Kuramitsu et al., 1980) and the inhibition of aggregation of streptococci by *Streptococcus pyogenes* LTA (Courtney and Hasty, 1991). In contrast, LTA is essential for the biofilm formation of *E. faecalis*, and an anti-LTA antibody interferes with its biofilm formation (Fabretti et al., 2006). D-Alanine acts as a key functional residue in LTA for the colonization of *S. aureus* (Gross et al., 2001). In addition, an LTA-deficient strain of *S. aureus*, Δ*ltaS*, only weakly formed biofilms compared to wild-type *S. aureus* (data not shown). Thus, extracellular LTA might be different from the membrane-anchored LTA that seems to be a positive regulator of bacterial biofilm formation.

Lp.LTA increased the release of AI-2 from *S. aureus*, and this release might mediate the inhibition of biofilm formation. In fact, AI-2 is a representative quorum-sensing signaling molecule known to inhibit biofilm formation in *S. aureus* and *Staphylococcus epidermidis* by inhibiting the transcription of *icaA* (Xu et al., 2006; Yu et al., 2012). We also observed the down-regulation of *ica* gene expression by Lp.LTA. Although the production of AI-2 is known to be mainly regulated by bacterial cell density, AI-2 has also been reported to be regulated by external stimuli such as Fe(III), NaCl, glucose, dithiothreitol, and flavonoid (DeLisa et al., 2001). Considering that LTA is actively released during the growth of Gram-positive bacteria, we tentatively suggest that LTA could function as a quorum to regulate AI-2 production in *S. aureus*, affecting its biofilm formation.

D-Alanine moieties in the Lp.LTA structure appeared to play a critical role in the inhibitory effect on *S. aureus* biofilm formation in that Lp.LTA deficient in D-alanine failed to inhibit *S. aureus* biofilm formation or to down-regulate *ica* gene expression. Although further studies are needed to clarify how D-alanine moieties contribute to the Lp.LTA inhibition of *S. aureus* biofilm formation, some possible explanations can be offered. First, a positive charge due to the existence of D-alanine may contribute to the inhibitory effect, since a positive charge has been implicated in the prevention of biofilm formation. For example, an increase in the degree of *N*-deacetylation (causing an increased positive charge) in chitosan-streptomycin conjugates augmented the anti-biofilm capacity of the antibiotics (Zhang et al., 2013). Similarly, positively charged liposomes are known to penetrate and inhibit biofilm formation by *S. aureus* and *P. aeruginosa* (Dong et al., 2015). Second, D-alanine may possibly interfere with *S. aureus* biofilm formation since D-amino acids are known to induce biofilm disassembly. Interestingly, the specific types of modulatory D-amino acids are dependent on the bacterial species. For example, *S. aureus* biofilm formation was inhibited by D-phenylalanine, D-proline, and D-tyrosine, while *B. subtilis* biofilm formation was inhibited by D-tyrosine, D-leucine, D-tryptophan, and D-methionine (Kolodkin-Gal et al., 2010). Nevertheless, considering the fact that D-amino acids are incorporated into peptidoglycan in place of D-alanine, it remains unclear whether D-alanine in the LTA structure affects biofilm disassembly as much as free amino acids do.

The inhibitory effect of Lp.LTA on *S. aureus* biofilm formation is not only due to competition with bacterial cell membrane-anchored LTA, but is also due to bacterial sensing and signaling in response to Lp.LTA because Lp.LTA could destroy the pre-formed biofilm. We showed that Lp.LTA could directly bind to *S. aureus*, where a certain type of receptor recognizing LTA may exist on the cell surface. For example, the two-component regulatory system may be involved, since ArlRS, LytSR, and SaeRS are directly involved in *S. aureus* biofilm formation, adhesion, and lysis (Fournier and Hooper, 2000; Sharma-Kuinkel et al., 2009; Liu et al., 2016). Therefore, further studies are needed to identify the potential receptor for LTA and its signaling pathways leading to the regulation of biofilm formation in *S. aureus*.

Previous reports have suggested that LTA negatively regulates autolysis in a variety of bacterial species. For example, LTA from *S. aureus* exhibits inhibitory effect on the autolysis (Suginaka et al., 1979; Fischer et al., 1981; Rice and Bayles, 2008). LTA from *S. pneumoniae* acts as an inhibitor of the homologous autolytic enzyme, an N-acetylmuramyl-L-alanine amidase (Holtje and Tomasz, 1975). Furthermore, LTA lacking D-alanine from *Lactobacillus lactis* increases the autolysis, through decreasing degradation of autolysin (AcmA) by extracellular protease (HtrA) (Steen et al., 2005). Therefore, one may expect that Lp.LTA inhibits autolysis in *S. aureus*. However, interestingly, Lp.LTA did not affect the viability or growth of *S. aureus* in our study. This is probably because the Lp.LTA was not derived from *S. aureus* that autolysis occurs, in the view of the fact that LTA is likely to inhibit the autolytic activity of homologous organism (Holtje and Tomasz, 1975). Remarkably, LTA from *S. aureus* and *L. plantarum* are structurally different, which leads to exhibiting different function (Kang et al., 2016). LTA from *S. aureus* DSM 20233 strain has 48 repeating units consisting of Gro-P with 70% D-alanine residue and contains only di-acylated glycolipid (DAG) (Morath et al., 2001), whereas LTA from *L. plantarum* L-137 strain has 96 repeating units consisting of Gro-P with 50% D-alanine residue and contains both DAG and tri-acylated glycolipid (TAG) (Hatano et al., 2015). Additionally, we also reported that LTA from *L. plantarum* KCTC10887BP strain has not only DAG but also TAG containing unsaturated fatty acids (Jang et al., 2011). Thus, the effect of each LTA on autolysis in *S. aureus* may be different due to its structural difference. Moreover, Sa.LTA targets AtlA, a major autolysin in *Staphylococcus* species (Biswas et al., 2006), whereas Lp.LTA targets Acm2, a major autolysin in *Lactobacillus* species (Fredriksen et al., 2012). Therefore, it is conceivable that Lp.LTA may not have affected the autolysis in *S. aureus*.

*S. aureus* causing chronic infections is associated with biofilm formation, which increases the antibiotic resistance. Thus, the combined therapeutics with antibiotics and anti-biofilm agent would be beneficial for the control of *S. aureus* infection. Indeed, we demonstrated that Lp.LTA potentiated the antimicrobial functions of various antibiotics. As an action mechanism, we hypothesized that Lp.LTA loosens the biofilm matrix by inhibiting bacterial aggregation and PNAG production, and facilitates penetration of antibiotics. In particular, inhibition of PNAG production in *S. aureus* by Lp.LTA appeared to be important for enhancing the antibiotic activity because EPS plays an important role in the resistance of bacteria by acting as a physical barrier against antibiotic penetration (König et al., 2001; Yamamoto et al., 2015). In addition, we also showed that Lp.LTA induced bacterial dispersion from pre-formed biofilm of *S. aueus*. Biofilm dispersing agents, such as Dispersin B, chitosan, and glycoside hydrolases, have been reported to release planktonic cells that are more susceptible to antibiotics than bacterial cells shielded by biofilm (Orgaz et al., 2011; Fleming et al., 2017; Waryah et al., 2017). Therefore, Lp.LTA may also act as a dispersing agent to potentiate antibiotic activity.

## Conclusion

The results of this study suggest that LTA is a major inhibitory molecule against *S. aureus* biofilm formation. Notably, biofilms are a major cause of various infectious diseases, but there are no biocompatible and effective therapeutics. Information provided by this study could be useful for the development of effective therapeutic agents to treat infectious diseases caused by *S. aureus* biofilms.

## Author Contributions

SHH and KBA conceived the idea and contributed to the discussion of the results followed by writing and reviewing the manuscript. SHH, KBA, and JEB designed the experiments, performed the experiments, and interpreted the data. C-HY provided critical comments and contributed to the discussion of the results followed by writing and reviewing the manuscript.

## Conflict of Interest Statement

The authors declare that the research was conducted in the absence of any commercial or financial relationships that could be construed as a potential conflict of interest.
